# Omega-3-Rich Oils from Marine Side Streams and Their Potential Application in Food

**DOI:** 10.3390/md19050233

**Published:** 2021-04-21

**Authors:** Mirian Pateiro, Rubén Domínguez, Theodoros Varzakas, Paulo E. S. Munekata, Elena Movilla Fierro, José M. Lorenzo

**Affiliations:** 1 Centro Tecnológico de la Carne de Galicia, Parque Tecnológico de Galicia, rúa Galicia No. 4, San Cibrao das Viñas, 32900 Ourense, Spain; rubendominguez@ceteca.net (R.D.); paulosichetti@ceteca.net (P.E.S.M.); 2 Department of Food Science and Technology, University of Peloponnese, Antikalamos, 24100 Kalamata, Greece; t.varzakas@uop.gr; 3 Complejo Hospitalario Universitario de Ourense, 32005 Ourense, Spain; elena.movilla.fierro@sergas.es; 4 Área de Tecnología de los Alimentos, Facultad de Ciencias de Ourense, Universidad de Vigo, 32004 Ourense, Spain

**Keywords:** PUFA-rich oils, seafood by-products, green extraction, functional foods, health benefits

## Abstract

Rapid population growth and increasing food demand have impacts on the environment due to the generation of residues, which could be managed using sustainable solutions such as the circular economy strategy (waste generated during food processing must be kept within the food chain). Reusing discarded fish remains is part of this management strategy, since they contain high-value ingredients and bioactive compounds that can be used for the development of nutraceuticals and functional foods. Fish side streams such as the head, liver, or skin or the cephalothorax, carapace, and tail from shellfish are important sources of oils rich in omega-3. In order to resolve the disadvantages associated with conventional methods, novel extraction techniques are being optimized to improve the quality and the oxidative stability of these high-value oils. Positive effects on cardiovascular and vision health, diabetes, cancer, anti-inflammatory and neuroprotective properties, and immune system improvement are among their recognized properties. Their incorporation into different model systems could contribute to the development of functional foods, with market benefits for consumers. These products improve the nutritional needs of specific population groups in a scenario where noncommunicable diseases and pandemic crises are responsible for several deaths worldwide.

## 1. Introduction

Today, more than ever, health and well-being play very important roles as the population wants to stay healthy in the face of the COVID-19 crisis [1]. For this reason, consumers have started searching for healthier options, trying to change their lifestyle and looking for more convenient foods [2]. In this sense, the nutrients and bioactive compounds found in food by-products can be excellent sources for the development of new food products to improve the health and well-being of consumers [3,4].

With rapid population growth, there is an increasing demand for food [5] but the associated waste generation and treatment costs will increase dramatically. This means that each part of the food supply chain, from food production to final domestic consumption, will generate substantial amounts of side streams [6]. Traditionally, the large quantity of food processing by-products has been considered industrial waste with no commercial value [7,8]. Therefore, effective mitigation actions and management strategies are urgently needed [5]. This scenario has forced the European Union to establish a new policy that employs new management actions that allow for the recovery of food biomass. Maintenance of these bio-wastes in the food chain can be achieved via the production of ingredients and biomolecules with high added value that could be used in the food and nutraceutical industries. Thus, the principles of a sustainable circular economy (green focus) are complied with [6]. In addition, the added value given to by-products allows for the reduction in waste according to the legislative frameworks developed by the European Union [9,10], which establishes recycling and recovery of waste as priorities in order to preserve natural resources and to protect the environment and consumer health by preventing or reducing the global impacts from the use of resources and by improving the efficiency of their use.

**Figure 1:**
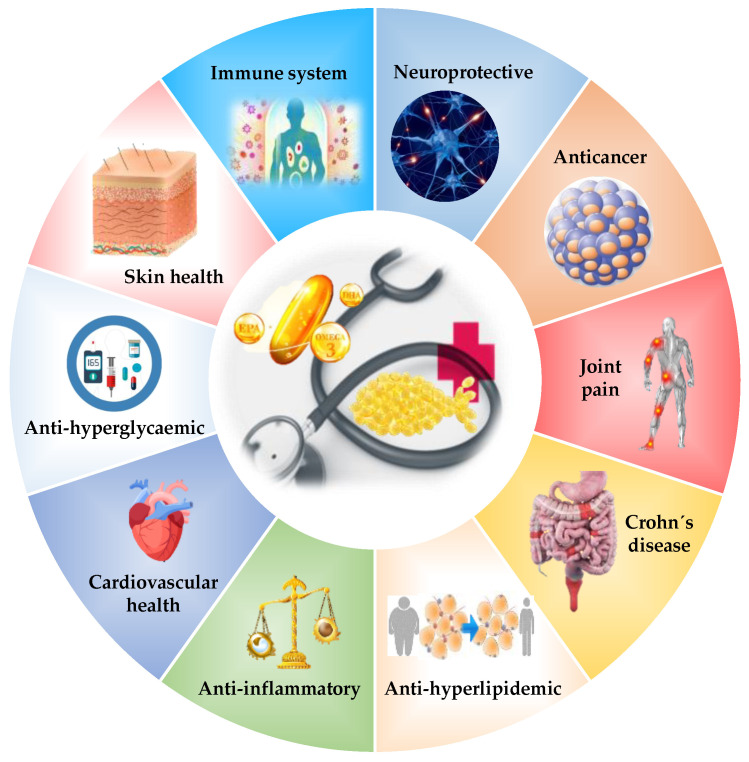
Health effects of omega-3 fatty acids.

Diet plays an important role in disease prevention and health promotion, since there are compounds such as long-chain *n*-3 polyunsaturated fatty acids (*n*-3 LCPUFAs) that the body cannot synthesize and that are essential for mammals [11]. In this group, eicosapentaenoic acid (C20:5*n*-3, EPA) and docosahexaenoic acid (C22:6*n*-3, DHA) are included. There are many studies that reflect the importance of these fatty acids in health. Anti-inflammatory properties; positive effects on cardiovascular and vision health, diabetes, cancer, immune system improvement, and development of the nervous system; reduced risk of Alzheimer’s diseases; and better digestibility are among their recognized properties [12,13,14]. It is important to note their ability to lower plasma triacylglycerol (TAG) (Figure 1). Fish oils are an important source of these healthy compounds [15]. In fact, these fish oils are often used as medication against lipid disorders (hypertriglyceridemia).

The quantity and quality of these fatty acids, known as omega-3, depend on many factors, including fish species, size, and age; gender; diet; habitat temperature; season; and extraction methods and conditions, among others [15,16]. In general, fish oils obtained from species reared in cold waters have higher contents of these fatty acids [17], although there is some controversy in this regard [18]. Ahmad et al. [14] demonstrated this by evaluating the fatty acid profile of common Australian seafood species (oily fish, *Salmo salar* and *Sardinops sagax*; prawns, *Penaeus plebejus*; and cephalopods, *Octopus tetricus* and *Sepioteuthis australis*). In addition, the contents are significantly higher in pelagic than in demersal species [18]. In this regard, oily fish such as tuna, salmon, sardine, and mackerel are important sources of these fatty acids [8,11].

During the filleting process, up to 60% of the fresh fish is cut off and generally treated as waste [19]. Several research studies have reported that these fatty acids are not only present in edible portions of fish and shellfish (subcutaneous tissue, belly flap, and muscle) but also in parts that are usually discarded (fishbone, gills, guts, head, liver, and skin) [14]. In this regard, high contents have been found in the mesenteric tissue, head, and liver. Hence, the production of omega-3 derived from marine side streams allow for recovery and the transfer of these important nutrients from the sea to the human food chain and promote economic growth, environmental protection, and human health. Thus, the aim of this review is to summarize the great potential of marine side streams as sources of *n*-3 LCPUFAs, presenting methods of recovery of these fatty acids, their functional properties, as well as their possible applications in food model systems.

## 2. Marine Side Streams as Sources of Omega-3 Fatty Acids

Seafood and the by-products generated from its processing are the main sources of *n*-3 LCPUFAs [8]. In fact, among the large number of bioactive compounds extracted from these products [6,20], EPA and DHA stand out. These are very valuable compounds that contribute to the significant market value of seafood exploited by the cosmetic, nutraceutical, and pharmaceutical industries [21]. In this regard, fish oils are supplements in great demand, mainly in Europe and in the United States [19], representing an 82.5% share in 2019 in terms of revenue, and it is expected to experience significant growth in the next years [22]. In Europe, this percentage was 22.4% in 2019, and it is expected to experience a significant growth in the next years, probably due to the aging population, who in Western European countries will be more than 20% of the world population [23].

The values presented in Table 1 show that marine side streams, especially viscera (liver and guts), have important contents of EPA and DHA. Therefore, they could be used for the extraction of oils rich in omega-3 fatty acids [24]. In this regard, most of the oils in the market are obtained from the liver of fatty species [25]. It can also be noted that, within omega-3 fatty acids, DHA is the most abundant, with contents up to 2–3 times higher than those observed for EPA [26].

As mentioned above, these fatty acids have an important role in human health. There are different ratios that reflect this effect. In this regard, both the *n*-6/n-3 and DHA/EPA ratios should be considered simultaneously. Several studies showed that lower DHA/EPA (1:2) ratios seem to be more effective at alleviating inflammatory risk factors [27]. These values depend on the fish species. For instance, the guts and head from Pacific Ocean perch comply with these recommendations, while these values were much higher in tuna.

**Table 1:** Omega-3 fatty acid content in different by-products of pelagic species (g/100 g of total fatty acids).

		Fishbone	Gills	Guts	Head	Liver	Skin	Ref.
Alaska Pink Salmon (*Oncorhynchus gorbuscha*)	C18:3*n*-3	-	-	0.95	1.10	-	-	[28]
	C20:5*n*-3	-	-	10.93	7.56	-	-	
	C22:5*n*-3	-	-	2.83	2.33	-	-	
	C22:6*n*-3	-	-	17.32	11.77	-	-	
	DHA/EPA	-	-	1.59	1.56	-	-	
Alaska Walleye Pollock (*Theragra chalcogramma*)	C18:3*n*-3	-	-	0.47	0.34	-	0.33	
	C20:5*n*-3	-	-	14.99	12.47	-	16.85	
	C22:5*n*-3	-	-	1.29	0.55	-	0.36	
	C22:6*n*-3	-	-	6.41	11.82	-	12.89	
	DHA/EPA	-	-	0.43	0.95	-	0.77	
Black rockfish (*Sebastes melanops*)	C18:3*n*-3	-	-	-	0.51	0.14	-	[29]
	C20:5*n*-3	-	-	-	9.92	4.43	-	
	C22:5*n*-3	-	-	-	1.65	1.38	-	
	C22:6*n*-3	-	-	-	9.21	4.78	-	
	DHA/EPA	-	-	-	0.93	1.08	-	
Black Sea Anchovy (*Engraulis encrasicholus*)	C18:3*n*-3	-	-	1.31	1.62	-	-	[30]
	C20:5*n*-3	-	-	6.93	10.97	-	-	
	C22:6*n*-3	-	-	18.88	21.34	-	-	
	DHA/EPA	-	-	2.72	1.95	-	-	
Pacific ocean perch (*Sebastes alutus*)	C20:5*n*-3	-	-	7.1	9.9	-	-	[25]
	C22:6*n*-3	-	-	3.3	4.7	-	-	
	DHA/EPA	-	-	0.47	0.48	-	-	
Sardine (*Sardinella lemuru*)	C20:5*n*-3	-	-	1.73	1.84	2.76	-	[31]
	C22:6*n*-3	-	-	11.87	15.95	12.97	-	
	DHA/EPA	-	-	6.86	8.67	4.70		
Salmon (*Salmo salar*)	C18:3*n*-3	-	-	1.14	-	-	-	[32]
	C20:5*n*-3	-	-	7.91	-	-	-	
	C22:5*n*-3	-	-	3.48	-	-	-	
	C22:6*n*-3	-	-	6.99	-	-	-	
	DHA/EPA	-	-	0.88	-	-	-	
Sea bream (*Sparus aurata*)	C18:3*n*-3	3.86	4.05	4.71	3.86	4.13	4.73	[8]
	C20:5*n*-3	2.77	1.92	1.83	2.78	1.91	2.03	
	C22:5*n*-3	2.00	1.42	1.55	2.00	2.04	1.66	
	C22:6*n*-3	4.58	4.09	3.51	5.00	4.90	3.98	
	DHA/EPA	1.65	2.13	1.91	1.80	2.57	1.96	
Sea bass (*Dicentrarchus labrax*)	C18:3*n*-3	3.77	2.70	3.30	3.69	2.00	3.10	[33]
	C20:5*n*-3	3.37	4.40	4.20	3.50	3.00	5.10	
	C22:5*n*-3	1.06	0.96	1.10	1.20	0.84	1.20	
	C22:6*n*-3	4.40	6.50	5.30	5.50	4.50	7.00	
	DHA/EPA	1.31	1.48	1.26	1.57	1.50	1.37	
Tuna (*Euthynnus affinis*)	C20:5*n*-3	-	-	2.71	1.48	1.70	-	[34]
	C22:6*n*-3	-	-	14.31	15.70	14.18	-	
	DHA/EPA	-	-	5.28	10.61	8.34	-	

-: not analyzed.

## 3. Extraction of Omega-3 Fatty Acids from Marine Side Streams

According to the information reported in the literature, only 5% of world fish oil production is used to extract omega-3 fatty acids destined for use as food ingredients or supplements [19]. The rest of the generated fractions are used for animal feed. However, the future trend is to reduce the levels of these ingredients (fishmeal and oils) in aquaculture diets, which will increase the available amount of these valuable essential fatty acids in human diet [35].

**Figure 2:**
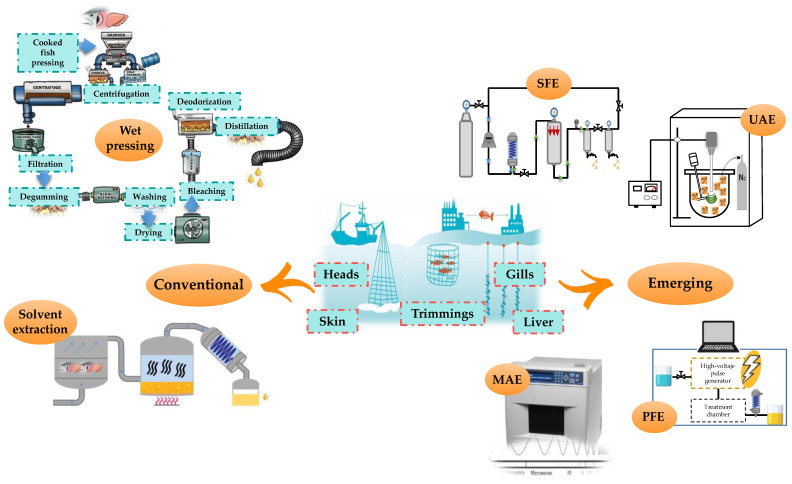
Methods for omega-3 fatty acid extraction from marine side streams. MAE: Microwave-assisted extraction; PEF: Pulsed electric fields; SFE: Supercritical fluid extraction; UAE: Ultrasound assisted extraction.

The conditions and extraction method affect the composition and the quality of the lipids extracted from marine processing side streams [36]. Therefore, selection of the method and its optimization are important considerations in producing omega-3 fatty acids with desirable characteristics. Some examples of these methods are presented in Figure 2.

### 3.1. Conventional Extraction

Different methods have been used for the extraction of omega-3 fish oil. Wet pressing is the method conventionally used for omega-3 fish oil. This extraction involves several steps: fish cooking, pressing, decantation, and centrifugation. Firstly, the fish is cooked and pressed. Then, the mixture is centrifuged to separate the fatty and aqueous fractions. The oil phase, which contains omega-3 fatty acids, is refined in several steps: neutralization, followed by bleaching, degumming or winterization, and deodorization [37]. This process allows us to improve the quality parameters of the oils, since it decreases the oil acidity, absorbs pigments or contaminants, separates the phospholipids, and removes smelly compounds. The resulting oil is characterized by ≅30% of omega-3 fatty acids, while other compounds such as cholesterol, omega-6 fatty acids, saturated fatty acids, oxidation products, and undesirable impurities comprise the rest of the oil components [19]. In order to increase the extraction yield of omega-3 fatty acids, a new step is usually included in this extraction process: molecular distillation. As a result, omega-3 ethyl esters constitute about 55% of omega-3 content produced.

Another possibility for the extraction of oil from fish and for processing by-products is solvent extraction. However, this method is not as common in the industry. The yield of this type of extraction depends on the solvent used. In this regard, Gulzar et al. [36] observed that isopropanol obtained the highest yields in the extraction of oil from Pacific white shrimp cephalothorax, followed by acetone, chloroform, and n-hexane (21.22, 18.23, 12.60, and 9.39%, respectively). These results could be improved with the use of solvent mixtures, which also depend on the marine side stream used. For mixtures of hexane, isopropanol at 1:1 (*v*/*v*) increased the yield to 25.44% in cephalothorax, while mixtures with acetone improved the yields in shrimp head and carapace [38,39].

### 3.2. Alternative Green Extraction Technologies

The yields obtained with conventional extraction methods are satisfactory; however, they also employ lengthy processes, have high solvent consumption, are mostly toxic, or use conditions that can degrade the quality of the extracted valuable compounds [6,40]. Therefore, it is necessary to explore viable and more sustainable processes. This has led the food industries and scientists to develop alternative processes according to the concept of “green” extraction, ensuring the purity and stability of these products [6]. Among the different green extraction processes, it is possible to mention microwave-assisted extraction (MAE), pulsed electric fields (PEF), supercritical fluid extraction (SFE), and ultrasound assisted extraction (UAE) [36,41,42].

#### 3.2.1. Supercritical Fluid Extraction (SFE)

In this regard, SFE is one of the technologies successfully used for the extraction of omega-3 fatty acids from marine side streams (Table 2). The application of this alternative green extraction method obtains nutritionally relevant ingredients (high content of omega-3 fatty acids) and reduces the associated environmental impact [43]. Tuna by-products such as the head and liver are products in which SFE is applied [44], since DHA can be up to 74% of the PUFA content [45]. Ferdosh et al. [46] found that conditions at 40 MPa, 3.0 mL/min CO_2_ flow, and 65 °C applied for 120 min on Longtail tuna (*Thunnus tonggol*) heads resulted in a PUFA-rich fraction with high quality and stability. EPA and DHA could also be obtained from the bones, scales, and skins of bigeye tuna (*Thunnus obesus*). Ahmed et al. [45] found that the application of 25 MPa, 40 °C, and a CO_2_ flow of 10 kg/h resulted in an oil with a combined EPA and DHA content in the range of 24.7–28.3%. Similar results were found by Létisse et al. [47] using sardine heads and tails, obtaining high yields (28% and 59% for EPA and DHA, respectively) and omega-3 contents (10.95% and 13.01% for EPA and DHA, respectively) with conditions at 30 MPa, 75 °C, 2.5 mL CO_2_/min, and 45 min.

The application of less drastic conditions could even improve extraction yields. In this way, yields greater than 96% of the total oil contained in hake off-cuts (*Merluccius capensis–Merluccius paradoxus*) were obtained by applying lower pressures (25 MPa) and temperatures (40 °C) and higher CO_2_ flows (10 kg CO_2_/h) [48]. These conditions produced oils with high omega-3 contents (6% and 14% for EPA and DHA, respectively), and less oxidation and impurities. The same was observed in the off-cuts of orange roughy (*Hoplostethus atlanticus*) and Atlantic salmon (*Salmo salar*) as well as on the liver from jumbo squid (*Dosidicus gigas*).

Shellfish is also an important source of bioactive components with potential health benefits. In this regard, krill, shrimp, and lobster appear to be alternative sources of omega-3 fatty acids [36]. This offers an alternative to these side streams, allowing us to obtain high-added value compounds from discards that sometimes represent up to 50% of the catch [49]. Amiguet et al. [49] optimized the extraction of omega-3 from the processing by-products (heads, shell, and tail) of Northern shrimp (*Pandalus borealis* Kreyer) with SFE [49]. The authors observed that a high selectivity for omega-3 contents (7.8% and 8.0% for EPA and DHA, respectively) was obtained with moderate pressure conditions (35 MPa at 40 °C and CO_2_ flow of 3–5 L/min for 90 min). Nguyen et al. [50] observed that the use of SFE also allowed us to obtain a safety product, since a reduction of toxic compounds was achieved, mainly arsenic and cadmium.

**Table 2:** Extraction of omega-3 (EPA and DHA)-enriched fish oils from marine side streams using SFE.

Side Stream	Source	SFE Conditions	Outcomes	Ref.
Caviar and viscera	Carp (*Cyprinus carpio* L.)	Temperature: 40, 50, and 60 °C Pressure: 200, 300, 350, and 400 bar CO_2_ flow: 0.194 kg/h Extraction time: 180 min	High yields (>50 g/100 g) in viscera, which are similar to those obtained with conventional methods	[51]
Head	*Thunnus tonggol*	Temperature: 65 °C Pressure: 40 MPa CO_2_ flow with ethanol: 3 mL/min Extraction time: 2 h	Co-solvent allowed to extract omega-3 after oil fractionations	[46]
Head, shells and tails	Northern shrimp (*Pandalus borealis* Kreyer)	Temperature: 40 °C Pressure: 35 MPa CO_2_ flow: 3–5 L/min Extraction time: 90 min	Lower yields (137 mg oil/g) than those obtained with solvent extraction. Higher fatty acid (795 mg/g), EPA (7.8%), and DHA (8%) contents	[49]
Heads and tails	Sardine	Temperature: 75 °C Pressure: 300 bar CO_2_ flow: 2.5 mL/min Extraction time: 45 min	Increased extraction yields: DHA (59%) and EPA (28%)	[47]
Liver	Rock lobsters (*Jasus edwardsii*)	Temperature: 50 °C Pressure: 35 MPa Continuous CO_2_ flow: 0.434 kg/h Extraction time: 4 h	Enrichment in PUFAs (DHA, EPA) vs. Soxhlet extraction Reduction of toxic heavy metals	[50]
Off-cuts	Hake (*Merluccius capensis*– *Merluccius paradoxus*) Orange roughy (*Hoplostethus atlanticus*) Salmon (*Salmo salar*)	Temperature: 313 K Pressure: 25 MPa CO_2_ flow: 880 kg/m^3^	Increased fish oil stability Reduction of impurities Co-extraction of some endogenous volatile compounds	[48]
Liver	Jumbo squid (*Dosidicus gigas*)			
Skins, scales and bones	Bigeye tuna (*Thunnus obesus*)	Temperature: 40 °C Pressure: 25 MPa CO_2_ flow: 10 kg/h	Recovery of 85.6, 83.2, and 87.7% of oil from skins, scales, and bones. EPA + DHA contents of 26.7–28.3%	[45]

#### 3.2.2. Other Alternative Green Analytical Techniques

Several research studies stated that UAE also enriches oils with high-added value compounds [39]. However, some studies indicate that this technology could enhance lipid oxidation, which is reflected in quality parameters as peroxide value, thiobarbituric acid reactive species (TBARS), and *ρ*-anisidine value. Therefore, some authors have applied UAE processes under nitrogen atmosphere to reduce the loss of quality in the obtained oil [52]. Another possibility would be the addition of antioxidant compounds during the extraction process [53]. As a result, DHA and EPA were retained more readily after the extraction (7.92 vs. 7.09 g/100 g and 10.65 vs. 9.84 g/100 g for EPA and DHA, respectively). Bruno et al. [54] evaluated the possibility of using UAE combined as a pre-treatment with enzymatic extraction for the extraction of oil from rohu (*Labeo rohita*) heads (Table 3). The results were satisfactory with regard to yields and omega-3 contents (0.88 and 0.13% for EPA and DHA, respectively).

PEF is another innovative technology that enhances the extraction of bioactive compounds and oils from marine side streams [6,55], being at the same time capable of maintaining oxidative stability and obtaining oils, probably due to inactivation of oxidative enzymes [36]. This technology used as pretreatment could be combined with others to improve the oil extraction yields. In this regard, the combination PEF–UAE resulted in increases up to 50% in lipid extraction from the cephalothorax of Pacific white shrimp [56].

**Table 3:** Extraction of omega-3-enriched oils from marine side streams using alternative green extraction technologies (MAE, PEF, and UAE).

Side Stream	Source	Extraction Conditions	Outcomes	Ref.
By-products	Catfish (*Pangasianodon gigas*–*Pangasianodon hypothalamus*)	MAE: 110 W, 1 min Enzymatic hydrolysis: Alcalase 2%, 2 h, 120 rpm	Pretreatments with MAE improved extraction yield and oil quality (lower lipid oxidation). Omega-3 contents 7.54 and 8.62% for EPA and DHA.	[57]
Cephalothorax	Pacific white shrimp (*Litopenaeus vannamei*)	PEF: 16 kV/cm, 240 pulses UAE: 80% amplitude, 25 min	Improved lipid extraction yield (30.34 g/100 g). Higher content of PUFAs (40.99 g/100 g lipids) and reduction of lipid oxidation. Omega-3 contents 8.20 and 10.39 g/100 g lipids for EPA and DHA.	[56]
Head	Rohu (*Labeo rohita*)	UAE: 20 kHz, 40% amplitude, 5–15 min. MAE: 200 W, 50 °C, 5–15 min Enzymatic hydrolysis: Protamex ratio of 1:100 (*w/w*), 2 h, 150 rpm, 55 °C	Pretreatments with UAE and MAE improved the extraction yield (67.48 and 69.75%, respectively). Omega-3 contents 0.86–0.88 and 0.13–0.16% for EPA and DHA.	[54]
Liver	Cobia (*Rachycentron canachum*)	UAE: 40 kHz, 1 h	Omega-3 contents 4.45 and 16.09% for EPA and DHA.	[58]
Viscera	Bighead carp	UAE: 400 W, 50 °C, 57 min	Extraction yield of oil reached 94.82%. Oil within standards of super fine crude fish oil.	[59]

MAE is another method used for the extraction of oil from fish processing by-products. However, it is not widely used and is usually combined with other traditional extraction methods to improve the extraction yields and to reduce lipid oxidation of the obtained oils [13]. Chimsook and Wannalangka [57] observed these positive effects when using MAE (110 W and 60 s) prior to enzymatic hydrolysis with alcalase to extract oil from catfish waste. Similar results were found by Bruno et al. [54] in the oil obtained from rohu heads using MAE as a pretreatment technology. 

According to the information reported in this section, it is plausible to conclude that these alternative methods are still under development and require additional research, with SFE being the most promising green extraction technology in obtaining oil from marine side streams.

## 4. Enrichment of Foods with Omega-3 Fatty Acids

Marine side streams are important sources of compounds with important technological, nutritional, and even functional properties, which when properly processed, can be reintegrated into the food chain. This entails the reduction of food processing waste and, therefore, the environmental impact of these types of industries. In this way, the food sector could contribute to a circular bioeconomy, making it possible to convert these by-products into marketable bio-products, generating new economic values and responding to society’s demand for a more efficient and sustainable environment. In addition, their incorporation into diets would contribute to improving its functional characteristics, thus diversifying the offer of the so-called “*Wellness Foods*”.

The fortification of food products with functional ingredients has gained increasing interest [36], since consumers are increasingly concerned about their health. In this regard, the demand for fish oil as a nutritional supplement in capsule form or as a food additive is increasing [60]. Within this promising strategy, food enrichment with fish oil provides functional products, which prevent cardiovascular, autoimmune, and inflammatory diseases and cancer, among others [61]. Bakery products, beverages, nutrition bars, dairy, and meat products are the main products fortified with this type of nutrient [62]. It is important to note that, when fortifying a product with oils rich in omega-3, the *n*-6/*n*-3 ratio must be balanced [63]. In addition, nutritional claims regulations establish that the required quantity of EPA + DHA to label a food as a “source of ω-3 fatty acids” must contain at least 40 mg of the sum of EPA and DHA per 100 g and per 100 kcal [64] while, to produce “high in ω-3 fatty acids”, at least 80 mg of the sum of EPA and DHA per 100 g and per 100 kcal is necessary.

The stability and effectiveness of the fortification, and the susceptibility of omega-3 to oxidation depend on the strategy to include fish oils in the product (directly, emulsified, structured gel, or nonencapsulated) [65]. In fact, the high unsaturation degree of these fatty acids makes them highly susceptible to oxidation and hydrolysis, which results in deterioration of the fortified product due to the appearance of strange flavors and fishy odors and causes consumer rejection [36,61,66]. This has led to the evaluation of different techniques to overcome these limitations. Encapsulation appears to be the most favorable strategy. The encapsulation of fish oils allows for protection against processing and storage conditions (exposure to oxygen and light, pH, temperature, storage time, etc.), masking the strong odors associated with these types of products [67]. Although there are several encapsulation techniques, spray drying is probably the most used technique today for microencapsulating sensitive bioactive compounds [36,68]. Some applications of fish oils rich in omega-3 fatty acids isolated from marine side streams are summarized in Table 4.

### 4.1. Bakery Products

Bakery and pasta products are some of the applications of these omega-3 fatty acids [69,70]. In this sense, shrimp oil rich in PUFA (2.15 and 6.20 g/100 g oil for EPA and DHA, respectively) was microencapsulated to increase the nutritional value of bread [71]. In contrast, fish oil extracted by conventional methods from hepatopancreas is characterized by high levels of rancidity. The microencapsulation using whey protein concentrate, sodium caseinate, and glucose syrup as wall material allowed for its conversion into a free-flowing powder. This microencapsulated fish oil (MFO) in powder form was introduced into the dough to fortify the bread at different levels (0, 1, 3, and 5%, *w*/*w*). This addition allows us to improve the technological properties of the product, increasing loaf volume, and decreasing chewiness (739.7 vs. 466.2 for the control and 5% MFO, respectively) and gumminess (759.3 vs. 503.9 for the control and 5% MFO, respectively), while no significant effect was observed in hardness (1362.2 vs. 927.9 g for the control and 5% MFO, respectively). The microstructure also revealed that the microencapsulated oil was perfectly embedded in the crumb. Regarding color, higher redness and yellowness were observed in the samples with MFO, probably due to the astaxanthin content. As expected, the microencapsulation protected the product against oxidation and sensory deterioration. However, these effects were dose dependent. In this way, the addition of concentrations up to 3% resulted in slight increases in the volatile (benzaldehyde) markers of oxidation (347 vs. 274 AU × 10^6^ for the control and 5% MFO, respectively) and no adverse effects in overall acceptability (6.80 vs. 7.03 for 3% MFO and the control, respectively).

**Table 4:** Omega-3 isolated from marine side streams and their potential use in foods.

Side Stream	Food Product	Dose and Incorporation	Storage Conditions	Outcomes	Ref.
Pacific whiteshrimp (*Litopenaeus vannamei*) hepatopancreas	Biscuits	Microencapsulates: 0, 3, 6, 9, and 12% (*w*/*w*)	12 days at 30 °C	No adverse effects on quality and acceptability up to 6% No marked change in EPA and DHA contents were noticeable after 12 days of storage Dark storage ensures its oxidative stability	[72]
	Bread	Microencapsulates: 0, 1, 3, and 5% (*w*/*w*)	3 days	No adverse effect on quality and sensory acceptability were observed up to 3% Oxidation took place in bread fortified with 5%	[71]
Sardine (*Sardinella brasiliensis*) head	Flour	20%	Final product	Increased content of DHA and EPA High acceptability index	[73]
Sardine (*Sardina pilchardus*) gills and viscera	Wheat flour-based chips	3.6% (*v/w*)		Improved nutritional (EPA 6.82% and DHA 8.27%) and health effects (antidiabetic, antihyperlipidemic, and histoprotective)	[74]
Sea bass (*Dicentrarchus labrax*) trimmings and small pieces	Fresh pasta	10%	90 days at refrigerated storage	Improvement of nutritional values. Decrease in hardness and cooking time Some sensory changes observed, mainly in fishy odor, and decrease in intensity of the typical yellow color	[75]
Cod liver	Cream cheese	Emulsion with CAS, WPI, or MPL: 1.3% (*w*/*w*)	20 weeks at 4.6 °C	Decreased oxidative stability (>5 weeks). MPL resulted in a more oxidative stable product	[76]
Pacific white shrimp (*Litopenaeus vannamei*) cephalothorax	Milk	Nanoliposomes: 0.05–0.2 g/100 mL	15 days at 4 °C	Half of EPA and DHA were bioaccessible for adsorption by the body in the gastrointestinal tract	[77]
Fish oil	Yogurt	Nanoencapsulates: 2% (*v*/*v*)	21 days at 4 °C	Higher DHA and EPA contents than yogurt with FFO. Reduction in acidity, syneresis, and PV. Sensory characteristics closer to control	[78]
		Microcapsules: 0.15% (*w*/*w*)	21 days at 6 °C	Improvement in health-promoting effect and consistency	[79]
Red salmon (*Oncorhynchus nerka*) heads	Strawberry-flavored yogurt	Microencapsulates: 2% (*w*/*v*)	30 days at 4 °C	No significant modification of physicochemical characteristics (pH, color, and WHC). After 4 weeks, EPA (2.11% of TFA) and DHA (1.72% of TFA) were the main fatty acids	[80]
Cod liver oil	Chicken nuggets	EPA + DHA in BFO and MFO nuggets was 150 mg/100 g MFO: 5% (*w*/*w*) BFO: 0.5%(*w*/*w*)	Final product	MFO provides lipid (<0.5 mg MDA/kg; hexanal 35.03 AU × 10^6^) and protein oxidation stability (≅3.6 nmol/mg). No effects of MFO on sensory attributes	[81]
	Cooked and dry-cured meat products	Mo (2.75% *w*/*w*) and Mu (5.26% *w*/*w*) emulsions	4 months at 0–5 °C	Enrichment in EPA and DHA: “source of ω-3 fatty acids”, without affecting main quality characteristics.	[82]

BFO: bulk fish oil; CAS: sodium caseinate; FFO: free fish oil; MFO: microencapsulated fish oil; Mo: monolayered emulsions; MPL: milk proteins and phospholipids; Mu: multilayered emulsions; TFA: total fatty acids; WHC: water-holding capacity; WPI: whey protein.

Similar results were observed when microencapsulated shrimp oil (0, 3, 6, 9, and 12%, *w*/*w*) was applied in biscuits [72]. In this case, sodium caseinate, fish gelatin, and glucose syrup were used as wall materials for the microencapsulation of hepatopancreas shrimp oil. The results obtained showed that a dose up to 6% avoids undesirable effects on the product. In this regard, the encapsulation allows us to protect EPA and DHA, maintaining the contents at the end of storage. Moreover, this technique also protects other easily oxidizable compounds, such as astaxanthin, from oxidation. However, again, the protective effect of the wall material allowed us to obtain slightly differences during storage. Regarding volatile compounds, the results suggest the convenience of storing cookies in dark conditions to avoid rancid odors resulted from oxidation reactions, which are accelerated in the presence of light (58 vs. 433 AU × 10^6^ for 2-heptenal in 6% MFO under dark and light conditions, respectively). At the sensorial level, the overall likeness scores were higher than those observed in control samples (5.52 vs. 7.12 for control and 3% MFO, respectively).

Another side streams that can be considered an important source of omega-3 fatty acids is the heads of sardines (*Sardinella brasiliensis*) [83]. These marine side streams are usually incorporated as oils, but they can also be used as flour (after being steam-cooked, ground, and dried). In this regard, de Oliveira et al. [73] evaluated the use of head sardines to increase the nutritional value of a flaxseed flour. The obtained flour was characterized by contents of EPA and DHA at 329.23 and 545.35 mg/100 g, respectively. To reach the daily requirements of EPA + DHA for the prevention of cardiovascular disease (250 mg/day [84]), one would need to take 28.58 g or 2 ½ tablespoons. In addition, the European regulations establish that, when making claims about food supplements and/or fortified foods, consumers must also be informed about the maximum supplemental daily intake of 5 g for both EPA and DHA in combination [84]. These levels were tested in the meals of industrial workers. Two meals were prepared: a control meal without omega-3 flour and a meal fortified with 1 or 2 tablespoons. The results showed that the levels of EPA (222.6–323.3%) and DHA (652.9–758.8%) increased considerably and that no significant differences were found between the addition of 1 or 2 tablespoon(s). Regarding the safety and acceptability of flour consumption by industrial workers, both evaluations were favorable. The sensory analysis results were even above the limits of acceptability for food products, 70% of the acceptability index, calculated by observing the global aspect × 100/9, where 9 is the highest score achieved on the hedonic scale [85].

A fish concentrate prepared from the by-products generated during the filleting process of sea bass (*Dicentrarchus labrax*) trimmings and small pieces were included in the formulation of fresh pasta (durum wheat and spelt) [75]. Moreover, the possibility of using an antioxidant (rosemary extract powder) with the fish concentrate was evaluated to avoid quality losses that could occur during storage. The addition of 10% fish concentrate resulted in the contents of EPA and DHA being 1.92–2.25% and 2.42–3.60% in the four type of pasta manufactured. These concentrations varied during storage probably due to lipid oxidation processes. In fact, the modifications in the fatty acid profile were more evident in the formulations without antioxidants, with DHA being the most affected. This behavior was confirmed by TBARS values and sensory attributes. Therefore, although the contents were adequate, the addition of antioxidants allow us to maintain the oxidative stability of the product, avoiding the appearance of defects in the product.

### 4.2. Dairy Products

The fortification of dairy products with omega-3 is a promising strategy for the supplementation of milk and fermented products (cheese, cream, milk deserts, and yoghurt), improving their nutritional value, stability, and health benefits. The fortification of these types of products can be carried out in the first stages of the food chain such as during feeding of the animals through the incorporation of fish oil into the feed of cows or goats [62].

As mentioned previously, encapsulation is the most efficient way to fortify foods with omega-3 fish oils. In fact, nanoliposomes have been used for milk fortification with omega-3 [86]. This lipid-based nanoencapsulation technique offers an attractive opportunity to integrate nutrients in foods, ensuring their stability and bioavailability [87]. In this regard, several studies have showed that the bioavailability of encapsulated omega-3 fatty acids is very similar to those observed with bulk fish oil [88]. The results found with the microencapsulated form improved the results in terms of sensorial attributes (without fishy or unpleasant flavors), oxidative stability, and retention of omega-3 during storage. The same outcome was observed when the milk was enriched with shrimp oil nanoliposomes at 10% (*v*/*v*) [77]. The analysis of EPA and DHA contents before and after in vitro digestion processes allowed us to assess the amount of fatty acids bioaccessible and absorbed in the gastrointestinal tract. In this case, 45.41 and 48.86 g/100 g were bioaccessible for EPA and DHA, respectively. In addition, fish oil encapsulated in liposomes allows for controlled release during digestion.

Yogurt is another product that can be fortified with omega-3, since it is one of the most consumed products worldwide and its matrix is very homogeneous [89,90]. Tamjidi et al. [79,91] evaluated the effect of the addition of microencapsulated fish oil containing 18% EPA, 12% DHA, and 200 ppm α-tocopherol on the physicochemical, rheological, and sensory characteristics of yogurt. The microcapsules produced by coacervation with gelatin-acacia gum allowed us to control the oxidation and rancidity of enriched yogurt. The addition of fish oil resulted in yellower samples probably due to the color of the gelatin used for the production of microcapsules. However, these differences were not significant with regard to color differences (ΔE) during storage. In addition, the obtained ΔE values were below the threshold to indicate noticeable color change (ΔE < 3 [92]). The sensory attributes were affected by the addition of fish oil, especially those related to aroma, flavor, and overall acceptability due to the unpleasant characteristics (off-flavors and odors) associated with this type of oil. We observed that the incorporation of lime juice allowed us to reduce these unpleasant attributes (4.00 vs. 5.30 for overall acceptance, respectively). Moreover, the apparent viscosity, acidity, and WHC were higher, while the gel strength was lower compared to the control samples.

Despite the generally positive effects found with the use of encapsulation, contradictory results were found by Horn et al. [76] in cream cheese fortified with cod fish oil. The authors compared the effect of the addition of neat oil or pre-emulsified oil. The emulsions prepared with 70% of cod liver oil-in-water and sodium caseinate, whey protein isolate, or a combination of milk phospholipids and milk proteins caused significant changes in the microstructure of the cream cheeses, which could be partly responsible for the oxidative stability during storage. Therefore, the authors proposed different strategies to achieve greater stability of the product during storage (more than five weeks). Among them, emulsion using a combination of proteins and phospholipids, or the inclusion of antioxidants enhanced the product stability.

### 4.3. Meat Products

Nowadays, meat industries are forced to reformulate their products due to consumer demands for healthier products [93]. Therefore, the incorporation of fish oils or concentrates in these products allows us to obtain products with important benefits for human health [94]. In this regard, the protector effect of microencapsulation as a vehicle for the incorporation of omega-3 fish oils in meat products was also observed in cooked and dry-cured meat products [82]. In this case, cod liver oil was encapsulated using monolayered and multilayered emulsions and incorporated in meat products at 2.75% and 5.26% (*w*/*w*), respectively. These concentrations allow us to reach the minimum amount required to label a food as a “source of ω-3 fatty acids” (40 mg EPA + DHA per 100 g and per 100 Kcal) [64]. The results obtained showed again that microencapsulation protects the oil from oxidative deterioration caused by processing and storage conditions, with a minimal effect in sensory acceptability. 

Pérez-Palacios et al. [81] evaluated the possibility of fortifying chicken nuggets by adding cod liver oils. The oils, stabilized with tocopherols (0.40 g/100 g oil), were characterized by EPA and DHA contents at 5.96% EPA and 25.83% DHA, respectively. This strategy is very important in ready-to-cook meat products, since the consumption of these products is expected to increase due to today’s lifestyles [95]. The oil was incorporated as bulk fish oil or microencapsulated with the aim to define the effect from the method of incorporation on the oxidative stability of the product. As expected, the evaluation of oxidation markers showed that samples produced with BFO displayed higher susceptibility to oxidation. This result was reflected in both primary and secondary lipid oxidation products. In the case of TBARS, the values obtained in BFO were higher than the limits of acceptability for meat products (0.6 mg MDA/kg) [96], while samples formulated with the microencapsulated oil allowed us to control these reactions (TBARS ≤ 0.5 mg/kg). These values were also reflected in the formation of volatile compounds used as lipid oxidation markers, which could explain the off-flavor resulting from the oxidation of fish oil [97]. In this way, the lowest values of hexanal were obtained in MFO samples (35.03 vs. 39.25 AU × 10^6^ for MFO and BFO, respectively). Together with hexanal, Jiménez-Martín et al. [98] observed the same effect in nonanal, also considered a lipid oxidation marker. These results were corroborated by those found by Aquilani et al. [99] in Cinta Senese burgers. Fortification with the same quantities of EPA + DHA in microencapsulated or bulk fish oil form resulted in a better stability of the first ones. This was reflected in the TBAR values and sensorial attributes. Moreover, the differences found were small with respect to control, showing even greater acceptability in samples refrigerated for 5 days.

Regarding protein oxidation, significant differences were also observed between samples, with control samples being those that showed the lowest values, followed by MFO and BFO (≅3.5, 3.6, and 3.8 nmol/mg protein, respectively). These differences mentioned above were also observed in sensorial attributes, especially in BFO samples, while MFO displayed values very close to the control samples. This similarity of the microencapsulated samples with the control samples could be related to the fact that the enrichment levels were lower (0.5% (*w*/*w*) and 0.15% *w*/*w* EPA + DHA) than those necessary to distinguish both types of samples (>2.5% (*w*/*w*) and >0.2% *w*/*w* of EPA + DHA) [100,101]. In view of these results, it is important to note that microencapsulation allows us to produce a viable product with similar attributes to control samples.

As commented upon before, the nutritional quality of meat products could be achieved by replacing animal fat with marine oils [93]. The incorporation of unencapsulated fish oils from cod (*Gadus morhua*) liver and salmon (*Salmo salar*) as partial substitutes (up to 15% *w*/*w*) of native fat in beef patties resulted in higher cooking losses and decreased hardness compared to samples that contained commercial encapsulated oils obtained from anchovies (*Engraulis ringens*) and sardines (*Sardinops sagax sagax*) [102]. Fish oils can also be incorporated in combination with gelling complexes or in the form of oil/water emulsions [103,104]. Domínguez et al. [105] studied the influence of partial pork backfat replacement by fish oil (50% and 75%) in liver pâté. Positive effects were observed on the nutritional values since the contents of *n*-3 LCPUFAs increased from 0.35 g/100 g, obtained in the control samples to 12.86 and 19.90 g/100 g in samples where pork backfat was replaced with 50 and 75% by fish oil, respectively. Effects in the technological parameters were also found since hardness and gumminess decreased as the content of fish oil increased. In contrast, an important increase was achieved on aldehyde contents, probably due to the different levels of polyunsaturation among batches. With this in mind, the most widely researched strategy to introduce fish oils in meat products is the use of encapsulation.

## 5. Conclusions and Future Perspectives

The nutritional deficiencies suffered around the globe, especially in the elderly group in some regions of the world, have increased the demand for functional foods and nutraceutical products, which reflects the special interest in fish oils rich in omega-3. The information collected in this review reflects the health benefits of these compounds but also shows that marine side streams offer a very important source of these fatty acids, especially in by-products with more fat such as viscera. The revalorization of these products has great benefits not only economically but also environmentally. On the one hand, the cost that their elimination would entail for the fish industries is reduced, and on the other hand, a great source of contamination posed by these side streams is avoided.

New technologies are being optimized with the aim of obtaining higher extraction yields and higher-quality oils rich in omega-3 from these marine sources. Although these alternative green methodologies do not have the same drawbacks as traditional methods, especially in the use of solvents and high temperatures, it is still necessary to optimize them to avoid the development of oxidative processes that would reduce the quality of the products obtained.

The unsaturation of these fatty acids makes them susceptible to oxidative processes that lead to nutritional and sensorial loss. This makes it necessary to search for alternatives solutions to solve this problem. In this regard, the combination of some of these technologies has been studied to increase the quality of the extracted omega-3 rich oils and, therefore, their beneficial properties, since their composition and structure remain almost intact. Another possibility would be to use anoxic environments during the extraction processes. Studies carried out under a stream of nitrogen show promising results and could be a solution to these deteriorations. However, the use of antioxidants seems to be the most efficient option. This strategy involves some controversy since synthetic antioxidants are often the most used for their efficiency and cost. However, the toxic effects linked to some of them make the use of natural antioxidants necessary, which motivates the search for new ones that could have their origin in agri-food co-products, thus complying with the principles of a sustainable circular economy (green approach).

From an application standpoint, it is important to note that the way omega-3-rich oils are incorporated into foods is a crucial factor. This affects the sensory attributes and the oxidative stability and, therefore, the quality of the final product. Encapsulation appears to be the most favorable strategy. In fact, several studies reflected that microencapsulation markedly increases the oxidative stability of omega-3 fatty acids. In addition, the lack of information about the bioaccessibility, bioavailability, and impact in gut microbiota of these new developed food products make it necessary to complete these studies in order to ensure their absorption.

It is plausible to conclude that marine side streams are a valuable source of omega-3 oils. Their application in food is a sustainable strategy that adds value to these parts often treated as waste while achieving important beneficial effects on the health of consumers. However, it is necessary to pay attention to the daily recommendations of these products for each population, since there is still no consensus about these among international organizations.

## Data Availability

The data presented in this study are available on request from the corresponding authors.
